# Positive Effects of α-Lactalbumin in the Management of Symptoms of Polycystic Ovary Syndrome

**DOI:** 10.3390/nu14153220

**Published:** 2022-08-06

**Authors:** Vincenzo Cardinale, Elisa Lepore, Sabrina Basciani, Salvatore Artale, Maurizio Nordio, Mariano Bizzarri, Vittorio Unfer

**Affiliations:** 1 Department of Medico-Surgical Sciences and Biotechnologies, Sapienza University of Rome, 00185 Rome, Italy; 2 R&D Department, Lo.Li. Pharma, 00156 Rome, Italy; 3 Department of Experimental Medicine, Section of Medical Pathophysiology, Food Science and Endocrinology, Sapienza University of Rome, 00161 Rome, Italy; 4 Oncology Unit, Gallarate-Busto Arsizio Hospital, ASST Valle Olona, 21013 Gallarate, Italy; 5 Department of Experimental Medicine, Sapienza University of Rome, 00161 Rome, Italy; 6 The Experts Group on Inositol in Basic and Clinical Research (EGOI), 00161 Rome, Italy; 7 Systems Biology Group Lab, 00161 Rome, Italy

**Keywords:** microbiota, α-Lactalbumin, metabolic alterations, mental health

## Abstract

To date, the involvement of α-Lactalbumin (α-LA) in the management of polycystic ovary syndrome (PCOS) refers to its ability to improve intestinal absorption of natural molecules like inositols, overcoming the inositol resistance. However, due to its own aminoacidic building blocks, α-LA is involved in various biological processes that can open new additional applications. A great portion of women with PCOS exhibit gastrointestinal dysbiosis, which is in turn one of the triggering mechanisms of the syndrome. Due to its prebiotic effect, α-LA can recover dysbiosis, also improving the insulin resistance, obesity and intestinal inflammation frequently associated with PCOS. Further observations suggest that altered gut microbiota negatively influence mental wellbeing. Depressive mood and low serotonin levels are indeed common features of women with PCOS. Thanks to its content of tryptophan, which is the precursor of serotonin, and considering the strict link between gut and brain, using α-LA contributes to preserving mental well-being by maintaining high levels of serotonin. In addition, considering women with PCOS seeking pregnancy, both altered microbiota and serotonin levels can induce later consequences in the offspring. Therefore, a deeper knowledge of potential applications of α-LA is required to transition to preclinical and clinical studies extending its therapeutic advantages in PCOS.

## 1. Introduction

α-Lactalbumin (α-LA) is a whey globular protein produced by the epithelial cells of the mammary glands [1] involved in lactose biosynthesis, which is essential for milk production. It is the predominant whey protein in human milk, and it has a high homology rate (72%) with the bovine isoform in the amino acid sequence [2,3].

α-LA also has high nutritional value due to its own aminoacidic building-blocks composition [4]. It is an important source of bioactive peptides and essential amino acids, including tryptophan (Trp), lysine, branched-chain amino acids (BCAAs), and sulphur-containing ones that play a central role in infant nutrition and human health [1]. While the relative abundance of Trp catches the eye for its effects on neurologic and behavioural outcomes, the plentiful cysteine content is pivotal for the synthesis of glutathione, which is an antioxidant and anti-inflammatory enzyme. Lastly, the leucine content plays an essential role in protein accretion and skeletal muscle growth [1].

For its physical characteristics of water solubility and heat stability, various food products add α-LA as supplemental protein. When orally administered, unlike all the other proteins that precipitate in the gastric environment, α-LA passes through the stomach unchanged, and it is a substrate of pancreatic enzymes (pepsin, trypsin and chymotrypsin) at the intestinal level. The bioactive peptides obtained from the proteolytic digestion of α-LA are responsible for multiple biological actions: antibacterial [5], anti-inflammatory [6], analgesic [7], antihypertensive [8], immunomodulatory and pro-apoptotic [9]. Preclinical and clinical studies report the antibacterial and immunostimulatory protective effects of such peptides by inducing phagocytic activity [5,10]. In fact, α-LA is a key agent in improving bacterial growth, gut microbiota and immunomodulation. By promoting the growth of several *Bifidobacterium* strains [11], these bioactive peptides from α-LA may further prevent infections related to pathogens like *Escherichia coli*, *Salmonella typhimurium*, *Staphylococcus aureus* and *Klebsiella pneumonia* [12], thus improving the chronic condition of altered microbiota.

Prebiotic activity like that of α-LA is further postulated as a novel approach for treating behavioural diseases and protecting mental health [13]. A preclinical study revealed that α-LA exhibited an anxiolytic effect by increasing the serotonin (5-HT) turnover in hippocampal areas [14]. In stress-vulnerable individuals, a diet enriched in α-LA improved mood and attenuated cortisol levels by increasing levels of Trp compared with the other large neutral amino acids (LNAAs), thus increasing brain serotonin content.

Therefore, taking the advantage of its aminoacidic profile and its multiple biological actions, α-LA finds extensive applications in human health, including those disorders exhibiting an altered microbiota among their pathological features, such as polycystic ovary syndrome (PCOS).

## 2. Microbial and Metabolic Association in the Pathogenesis of PCOS

PCOS is an endocrine and metabolic disorder characterized by complex etiology and pathophysiology. Recent studies revealed a strong association in patients with PCOS between changes in gut microbiota and some clinical–metabolic parameters, suggesting that microbiota alterations may play a critical role in the pathogenesis of this syndrome [15,16].

Generally, the term “microbiota” refers to all microorganisms (bacteria, archea, protozoa, fungi, etc.) populating and colonizing different surfaces of the human body, such as skin, mouth, gastrointestinal system, and vagina [17]. The composition of each community is site-specific and depends upon several host-derived factors: available nutrients, hormone levels, host genetics, and age [18,19]. A great amount of evidence suggests that alterations in microbiota composition, referred to as dysbiosis, can impact crucial processes, resulting in chronic diseases such as inflammatory bowel disease, type 2 diabetes, obesity and ultimately PCOS [20,21,22,23,24,25,26,27]. Indeed, the gut microbiota itself is involved in the synthesis of several hormones, vitamins and neurotransmitters [28,29,30].

PCOS is a heterogeneous pathological condition mainly characterized by three clinical features, including (i) menstrual disorders, (ii) hyperandrogenism, and (iii) polycystic ovary [31]. However, beyond these classic signs, most affected patients exhibit gastrointestinal dysbiosis [32], which is one of the mechanisms triggering the development of the PCOS. In line with this, the microbiological theory for the pathogenesis of PCOS precisely arises from this gut dysbiosis, which increases the production of ovarian androgens and interferes with physiological follicle development, leading to insulin resistance and intestinal inflammation [32]. PCOS-associated gastrointestinal dysbiosis consists of decreased α-diversity and changes in the β-diversity of microbiota. Such alteration reflects changes in the composition of the microbial community, with differences in the relative abundance of specific phyla like *Firmicutes* and *Bacteroidetes* [33,34]. A preclinical study in a PCOS-like mouse model evaluated the influence of *Bacteroides vulgatus* on PCOS pathogenesis: oral gavage with *Bacteroides vulgatus* in such mice induced a phenotype of insulin resistance and disrupted estrus cycle and ovarian morphology [35]. Researchers confirmed these findings by revealing a significant increase in *Bacteroides vulgatus* also in patients with PCOS compared with healthy controls [35].

Obesity and high-fat–low-fiber diets represent two key conditions in determining gut dysbiosis [27,32], as they can induce a shift composition of the microbiota in favor of pro-inflammatory bacteria. This modification indeed weakens intestinal tight junctions and the mucous barrier, and increases the permeability of the gut mucosa, exacerbating the systemic dissemination of inflammatory mediators and causing the activation of the immune system [32]. Ultimately, this process interferes with insulin receptor function, determining hyperinsulinemia, which can increase ovarian androgens and prevent the development of normal follicles. This leads to the classic PCOS pathological features including menstrual disorder, hyperandrogenism, and polycystic ovary, supporting the so-called microbiological theory of PCOS, “dysbiosis of gut microbiota” (DOGMA) [32]. According to the DOGMA theory, both diet-induced and obesity-induced dysbiosis can trigger the development of PCOS [27,32,36]. Indeed, obesity and weight gain are common conditions in women with PCOS since visceral adiposity is associated with increased insulin resistance as well as metabolic and reproductive alterations [37,38]. Patients with PCOS exhibit a higher prevalence of obesity compared with women without PCOS [39], with incidence ranging from 30% to 70% [40]. Previous studies reported that obese women with PCOS had more deleterious metabolic disorders, including insulin resistance, dyslipidemia [37,41], glucose intolerance and increased long-term risk of cardiovascular disease [42], hypertension, depression and non-alcoholic fatty liver disease (NAFLD) [43], compared to women with obesity or PCOS alone. Rui Liu and colleagues confirmed that conditions of obesity and PCOS shared a similar dysbiosis of gut microbiota, and in particular, obese women with PCOS exhibited more serious gut dysbiosis [44]. Some Gram-negative bacteria belonging to the genera *Bacteroides* and *Escherichia/Shigella* are significantly increased in the gut of obese women with PCOS. In addition, the lipopolysaccharide (LPS) produced by Gram-negative bacteria may induce obesity, insulin resistance and inflammation in LPS-infused mice [45].

Different studies further demonstrated that a strong relationship between microbiota and sex hormone levels contributes to PCOS pathogenesis [46] and that changes in the gut microbiota of patients with PCOS correlate with hyperandrogenism and features like hirsutism and high testosterone levels [36,47,48]. The high abundance of *Prevotella* in patients with PCOS positively correlates with androgens’ levels, especially testosterone and androstenedione [34,49], while it negatively correlates with estradiol concentrations [50]. Also, the abundance of *Kandleria* correlates with the circulating androstenedione concentration [51], suggesting that levels of androgens may be crucial for shaping gut microbiota in the development of this syndrome [33].

In addition, previous evidence demonstrated the shifts of gut microbiota in mouse models of letrozole-induced PCOS compared with wildtype ones [52]. The fecal microbiota transplantation (FMT) from letrozole-induced PCOS mice to germ-free ones indicated a strict relationship between gut microbiota and host’s estrus cycles, ovarian morphological changes and sex hormone levels [46]. As an aromatase inhibitor that induces hyperandrogenemia, letrozole treatment correlates with significant changes in the abundance of specific *Bacteroidetes* and *Firmicutes*. In particular, *Bacteroidales* decrease while *Clostridiales* increase in letrozole-induced PCOS mice compared with wildtype ones. In addition, *Lactobacillus*, *Ruminococcus* and *Clostridium* were lower, while *Prevotella* was highly present in PCOS rats compared with healthy animals. On the contrary, the FMT or the administration of *Lactobacillus* from healthy rats in letrozole-induced PCOS mice improved the oestrus cycle with decreased androgen biosynthesis and restored ovarian morphology. This transplantation of *Lactobacilli* or faecal microbiota from healthy rats improved the PCOS phenotype, reducing androgen biosynthesis [46] and indicating that *Lactobacilli* transplantation correlated with oestradiol and estrone levels. Indeed, both *Lactobacilli* and *Bifidobacteria*, which are involved in enhancing immunity and nutrient absorption, were significantly reduced in patients with PCOS [46,53]. These data associate the dysbiosis of gut microbiota with the pathogenesis of PCOS and confirm that interventions on microbiota positively affect PCOS features.

Notably, the gut microbiota is not the only altered tissue niche in women with PCOS. Female gut and genital tract microbiota are in an intimate cross talk: a healthy vaginal microbiome evolves through a continuous translocation of species from gut to vagina [54], impacting host immunological and metabolic homeostasis. In particular, both vaginal and cervical canals in patients with PCOS exhibit a great heterogeneity of microbiota composition with a significant reduction of *Lactobacillus* and a concomitant increase of pathogenic taxa, including *Gardnerella vaginalis*, *Chlamydia trachomatis* and *Prevotella* [34].

In a case-control study, Hong and colleagues pointed out that PCOS displays alterations in vaginal microbiota, including α- and β-diversity parameters. They observed vaginal dysbiosis characterized by a reduction in *Lactobacillus*, in particular *Lactobacillus crispatus*, which is a beneficial bacterium in the vaginal niche, in PCOS condition [55]. Homogeneous *Lactobacillus*-dominated microbiome and reduced species diversity represent hallmarks of a healthy female reproductive tract [56]. The α-diversity strongly decreased in women with PCOS, and alterations in the *Streptococcus genus* may impact pathological changes of the syndrome, including the female reproductive endocrine environment [57].

Overall, the emerging awareness about the crucial role of microbiota in this syndrome opens novel strategies for efficaciously re-balancing the gut and vaginal microbiota. One possibility is to improve metabolic and hormonal PCOS symptoms by targeting microbiota using molecules with prebiotic activity. On these premises, one could hypothetically extend the use of α-LA in the management of PCOS. Even though other options including oatmeal or wheat germ have positive effects as prebiotics [58,59,60], in the management of women with PCOS, α-LA already plays a positive role that can be expanded toward a prebiotic activity. To date, the use of α-LA in this syndrome is just considered in association with inositols [61,62], aiming to improve their intestinal adsorption and overcoming the common problem of inositol resistance occurring in some of these patients [63,64,65]. The study by Montanino and colleagues indicated that adding 50 milligrams of α-LA to 2 g of myo-Inositol twice a day restored ovulation in 86% of inositol-resistant women with PCOS.

Using α-LA as a multidisciplinary or multifactorial approach still remains a knowledge gap to bridge. As the clinical picture of this syndrome can vary over the life span in the same patient, making tailored approaches and recommendations remains the best option for treating such women [66].

## 3. α-Lactalbumin for Improving Dysbiosis

Given that the microbiota is involved in PCOS pathogenesis, approaches aiming to recover dysbiosis are becoming increasingly important in the treatment protocols of PCOS. The recommendations of the 2018 International Guidelines encouraged lifestyle-based strategies among first-line managements, with the attempt to restore eubiosis in patients with PCOS [67].

Women with and without PCOS exhibit such different baseline dietary intakes that an unhealthy diet in case of PCOS may contribute to worse insulin resistance, hyperandrogenism and inflammatory status [68]. Evidence in the literature revealed that most women with PCOS consume an improperly balanced diet and that instead following a specific diet may improve clinical symptoms. For instance, Paoli and colleagues demonstrated that overweight women with PCOS under a modified ketogenic diet improved both their metabolic (body mass index, insulin resistance) and hormonal (LH, testosterone, estradiol progesterone) parameters [69]. Nutritional management is crucial in women with PCOS, and a healthy diet is useful for counteracting metabolic alterations; however, the assumption of prebiotics helps to recover the dysbiosis and low intestinal adsorption characterizing this pathology. Indeed, since the role of intestinal microbiota in PCOS pathogenesis, the use of microbiota-targeted strategies became increasingly important. Due to its prebiotic activity, α-LA may control the establishment of a proper intestinal flora, preventing or recovering dysbiosis and related metabolic effects. A recent meta-analysis evaluating 12 randomized controlled studies indicated that the use of some bacterial strains, including those stimulated by α-LA (*Lactobacillus acidophilus*, *Bifidobacterium short*, *Bifidobacterium longum* and *Bifidobacterium infantis*), is associated with a significant improvement in the glycated haemoglobin and HOMA-IR (Homeostatic Model Assessment for Insulin Resistance) in patients with type 2 diabetes [70], suggesting the positive effects of α-LA in restoring metabolic parameters. Indeed, *Bifidobacteria* and beneficial gut flora stimulated by α-LA produce short-chain fatty acids (SCFAs), such as butyrate and acetate, which may positively influence glycemia and improve insulin resistance [71].

The probiotic supplementation with *Lactobacillus acidophilus*, *Lactobacillus casei* and *Bifidobacterium bifidum* for a period of 12 weeks strongly decreased weight and body mass index in women with PCOS, also improving levels of glycemia, triglycerides and very-low-density lipoproteins (VLDL) [72]. An 8-week-supplementation of *Bifidobacteria* and *Lactobacilli* induced similar beneficial effects with a significant decrease in plasma glucose and insulin levels [73]. Further evidence comes from the study of Rashad and colleagues in which supplementation for 12 weeks with a probiotic containing *Lactobacillus delbruekii* and *Lactobacillus fermentum* significantly reduced HOMA-IR, with an additional improvement of lipid profile [74].

In addition to positive effects on metabolic profiles, Ghanei and colleagues demonstrated that the probiotic administration of *Lactobacillus acidophilus*, *Lactobacillus plantarum*, *Lactobacillus fermentum*, and *Lactobacillus gasseri* improved inflammatory processes in women with PCOS after 12 weeks of treatment [75].

Consistent with these results and considering the prebiotic effect of α-LA, studies on mouse models reported that hydrolysed α-LA exerted protective effects against insulin resistance and inflammatory status in adipose tissue of mice fed with a high-fat diet (HFD). In detail, they observed that the supplementation with α-LA significantly reduced body weight, blood sugar, insulin level and HOMA-IR, decreasing pro-inflammatory factors such as interleukin-6 (IL-6), tumour necrosis factor-α (TNF-α) and monocyte chemoattractant protein-1 (MCP-1) in HFD-fed mice [76]. Later studies in murine models expanded these results, demonstrating that diets enriched in α-LA and its hydrolysates (LAH) may positively affect the taxonomic composition of microbiota, properly increasing *Bifidobacterium* and *Lactobacillus* populations. These studies reported the relative abundance of *Bacteroidetes* and the significant increase of *Bacteroidetes*/*Firmicutes* ratios in α-LA or LAH groups compared with the HFD group [77,78]. This shift in microbiota composition toward probiotic bacteria led to an increased production of SCFAs such as acetate, propionate and butyrate [36,78], resulting in an improved gut mucosa barrier and reduced endotoxemia in the α-LA and LAH groups compared with the HFD group [77].

Overall, treatments with probiotics and prebiotics exhibiting beneficial effects on metabolic and hormonal parameters in women with PCOS confirmed the possibility of considering them new additional treatment option for PCOS [16]. Clinical evidence supporting α-LA’s prebiotic effects and its ability to recover altered microbiota composition, insulin resistance and intestinal inflammation add novel perspectives to its use for improving metabolic phenotypes in patients with PCOS. In other words, α-LA can address the need for a multidirectional therapeutic approach in patients with PCOS, considering both its effect on inositol resistance and its wide range of activity managing several aspects of the syndrome.

### α-Lactalbumin Positively Affects Maternal Dysbiosis Shaping Neonatal Microbiota

Healthy vaginal and gut microbiota play a crucial role also with respect to the mother-to-child microbiological transfer and the resulting neonatal outcomes. Considering its role in stimulating the initial colonization of the infant gut, the mother’s microbiota is one of the factors shaping neonatal microbiota [79]. Alterations of maternal gut microbiota resulting from obesity, gestational diabetes mellitus (GDM), poor nutrition, PCOS or even stress may ultimately influence the microbial composition to which the foetus is exposed in utero, leading to long-term changes in foetal gut functions and compromising the offspring’s metabolic profile. For instance, Ponzo and colleagues reported that GDM is associated with specific changes in gut microbiota that may influence offspring microbiota [80,81,82]. As a matter of fact, a study from Gower and colleagues revealed that specific taxa associated with GDM can be transmitted to offspring microbiota, thus increasing the risk for the offspring to develop dysmetabolic diseases such as obesity or diabetes mellitus [83] compared with offspring of normoglycemic women [82,84,85].

In addition, Collado and co-workers compared the microbiota of infants born respectively from overweight and normal-weight mothers [86]. Infants born from overweight mothers exhibited increased *Bacteroides* and *Staphylococcus* at both 1 and 6 months of age. This observation reflects the altered maternal microbiota composition during pregnancy [87] and suggests a role of mother’s microbiota in shaping the early composition of neonatal microbiota. Indeed, higher body weight and body mass index of mothers correlated with higher abundance of *Bacteroides*, *Clostridium*, and *Staphylococcus* and lower concentrations of the *Bifidobacterium* group.

Considering the prevalence of obesity in women with PCOS and the more serious gut dysbiosis occurring in these patients compared with obese women without PCOS, altered gut microbiota can deeply influence the pregnancies of those women with the syndrome. In particular, as neonates largely acquire their microbiota from their mothers, one could argue that neonates from mothers with PCOS acquire an altered microbiota which reflects the maternal composition [88]. The prenatal exposure to maternal hyperandrogenic milieu in pregnant women with PCOS results in increased androgen secretion, insulin resistance and weight gain at birth, supporting the hypothesis of a transmission of PCOS dysbiotic microbiota from the mother to neonates [89,90]. Gulan and colleagues came up with the same findings using a rat model of PCOS. Additionally in their experiments, exposure to androgens in utero resulted in the dysbiosis of the intestinal microbiota and metabolic disorders of new-born rats [91]. Interestingly, a clinical study reported that the cord blood of babies born from mothers with PCOS had higher levels of androgens [92], while another study indicated higher concentrations of testosterone in the umbilical vein of female neonates [93]. In addition, the increased serum androgen levels correlate with shifts in the microbiota in women with PCOS [94,95]. This means that gut microbiota may exert a physiological role in the androgen metabolism and that a bidirectional interaction exists between maternal hyperandrogenemia and microbiota. In summary, mothers’ androgen levels are influenced by their microbiota either directly or indirectly, and, at the same time, these androgen levels may condition the composition of maternal microbiota, spreading the effects to the offspring [94,96,97].

As microbiota alterations in pregnant women are related to long-term changes in the offspring microbiota [98,99], interventions in maternal nutrition before and during pregnancy may positively impact foetal growth and development. At the same time, pregnancy-related modifications in the composition of maternal gut microbiota influence nutrient adsorption as well as foetal gut colonization and metabolic tissue development, determining the exposure to risk factors involved in the development of chronic diseases in the offspring [100,101].

Considering the beneficial effects derived from recovering intestinal eubiosis, α-LA may act on multiple aspects, both recovering gastrointestinal dysbiosis and preventing alterations occurring in the offspring. Optimizing nutrient intestinal adsorption, α-LA improves beneficial effects of the natural molecules of inositols on the above-mentioned pathological conditions like PCOS, GDM, insulin resistance and obesity [102,103,104,105,106]. In addition, directly impacting the intestinal niche, α-LA may stimulate microbiota remodelling, thus preventing and counteracting microbiota-dependent metabolic disorders in the offspring. Indeed, α-LA may promote the physiological shaping and maturation of gastrointestinal tract during foetal development and, concomitantly, restore the integrity of the intestinal niche without exposing babies born from women with PCOS to metabolic risks.

Given the centrality of the intestine in influencing the state of health/disease of the whole organism, as well as the neonates, and considering the high frequency of gastrointestinal dysbiosis in women with PCOS, the recovery of altered intestinal microbial composition is a crucial aspect in the management of pregnant women with PCOS. Therefore, the possibility of early recovering the microbiota of the offspring and of preventing consequences later in life, remains of potential great interest.

## 4. Intestinal and Mental Health: Working Hypothesis for a Positive Effect of α-Lactalbumin on Mood

Several studies highlight the connection between intestinal eubiosis and mental health. Microbial populations inhabiting the intestinal niche contribute to the synthesis of vitamins, minerals and peptides that may communicate with the brain. Alterations in gut microbiota can influence the secretion of such vitamins and minerals, whose depletion correlates with the pathogenesis of depression and other mental disorders like anxiety and aggression.

A large amount of studies reported high psychological distress in women with PCOS compared with healthy populations [107]. In particular, depressive mood is a frequent and common aspect in patients with PCOS. Features linked to this syndrome like infertility, weight gain, hirsutism and acne may negatively impact the mood and psychological well-being of these women [108]. Besides the association with altered gut–brain axis, depressive symptoms in PCOS correlate with reduced serotonin levels. Gut dysbiosis causes changes in serotonin secretion, affecting brain development, stress response and emotional activities including anxiety and depression [108].

Evidence in literature demonstrated that both mouse models of PCOS and patients suffering from this syndrome are characterized by low levels of serotonin. Chaudhari and colleagues reported that mice with PCOS exhibited lower levels of serotonin in several regions of the central nervous system compared with wildtype animals [109]. Shi and colleagues also described significantly lower levels of both serotonin and its main metabolite, 5-Hydroxyindoleacetic acid (5-HIAA), in infertile patients with PCOS [110]. Interestingly, lower serum levels of both serotonin and dopamine metabolites correlate with an altered secretion of the luteinizing hormone (LH) in PCOS patients [110]. Indeed, previous evidence reported that serotonin is involved in the secretion of pituitary LH by stimulating its release in young female rats [111]. Studies in murine models of PCOS assessed that brain downregulation of both serotonin and its main metabolite, 5-HIAA, inhibited murine reproductive function and subsequently decreased hypothalamic GnRH content [112]. This finding provided new insights into the classic concept of PCOS, highlighting the correlation between hormonal levels and affective disorders. Preclinical and clinical studies from Lokuge and colleagues reported that estrogens may regulate serotonin pathways at various levels by increasing serotonin availability and decreasing its breakdown [113]. Therefore, the low proportion of both serotonin and its metabolites in women with PCOS is in line with the typical lower levels of estrogens described in such patients.

Interestingly, α-LA dietary supplementation significantly improves serotonin synthesis and its release from serotonergic neurons [14], with positive effects on mood depression and anxiety. Diets enriched in α-LA increased the plasma ratio between Trp and other LNAAs up to 48% compared with other whey proteins. Being Trp the precursor of serotonin, this increased ratio correlated to higher brain serotonin content [114]. In healthy subjects, the ingestion of α-LA induced a marked increase in Trp concentration [115] for at least 4 hours and a change in brain Trp concentrations and serotonin synthesis with respect to different dietary proteins [116].

Overall, such evidence clearly depicts that α-LA supplementation increases levels of Trp and serotonin, positively affecting depressive mood and anxiety, which are common features in patients with PCOS.

The beneficial effect of α-LA on serotonin levels is gaining importance especially for pregnant women with PCOS or women seeking pregnancy. Serotonin acts as a regulator of the physiological development of brain [117], impacting synaptic plasticity and maintenance. Indeed, during embryogenesis, serotonin signalling plays a pivotal role in the cascade of events leading to brain development and structural changes. The exposure to both low and high serotonin levels during the perinatal period can lead to behavioural alterations in adulthood [118] due to a reduction in serotonergic signalling. While Trp depletion may directly impair the development of the serotonergic system [119], excessive serotonergic signalling may reduce serotonergic activity/development through negative feedback. Research on animal models confirmed that female rats fed a low-Trp diet during the early developmental age (weaning age-3 weeks) exhibited a delay in growth, ovarian function and sexual maturation in the offspring [118,120,121]. Other studies demonstrated that increasing serotonin levels through exposure to selective serotonin reuptake inhibitors (SSRIs) during perinatal life may lead to emotional deficits in adulthood (e.g., increased anxiety and depressive-like behaviours) [18,118].

These findings confirmed that serotonin is crucially involved in neurodevelopment and that changes in its levels during the perinatal and postnatal period [122] can lead to multiple and long-lasting negative effects [119,123]. Therefore, considering the low levels of serotonin occurring in women with PCOS, the intake of α-LA in these patients may significantly support the physiological levels of this neurotransmitter during pregnancy, avoiding the risk of exposing the foetus to altered brain developmental processes with long-term consequences.

## 5. Conclusions

**Figure 1:**
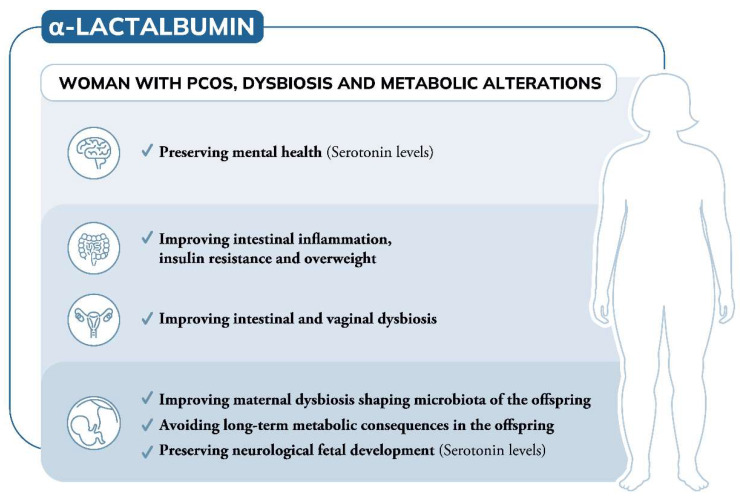
**α-Lactalbumin role in the management of women with PCOS, dysbiosis and metabolic alterations**. Acting on different aspects, α-Lactalbumin (α-LA) induces beneficial effects in women with PCOS exhibiting dysbiosis and metabolic alterations. Considering its prebiotic activity, α-LA may improve intestinal and vaginal dysbiosis recovering PCOS related metabolic alterations. In addition, due to its content in tryptophan, α-LA may preserve mental health by raising serotonin levels. Regarding women with PCOS seeking pregnancy or those pregnant, α-LA, by improving maternal microbiota, shapes the microbiota of the offspring, avoiding exposing the foetus to long-term metabolic consequences and altered neural development.

The specific amino acidic composition and its physical properties make α-LA a promising additional dietary supplement in the management of PCOS. Indeed, being a source of the aminoacidic precursor of serotonin and acting as prebiotic molecule, α-LA may improve both serotonin levels and intestinal eubiosis, adding new insights for its application in the management of PCOS (Figure 1).

Due to its prebiotic effect, α-LA may recover altered microbiota composition, reducing related insulin resistance, overweight and intestinal inflammation occurring in patients with PCOS. These actions promote the recovery of gut and vaginal dysbiosis, which are frequent conditions in patients with this syndrome.

By restoring the intestinal eubiosis, α-LA further contributes to preserving mental health, positively affecting several aspects related to PCOS such as depressive mood correlated to low levels of serotonin in affected patients.

Moreover, in pregnant women with PCOS or other metabolic alterations, altered gut and vaginal microbiota, along with low levels of serotonin, are crucial for shaping microbiota colonization in the offspring, determining long-term metabolic consequences. Therefore, keeping in mind its prebiotic role and its ability to recover altered microbiota both in women with PCOS and in the offspring, α-LA may improve maternal intestinal dysfunctions and permeability, reducing long-term metabolic consequences for both the mother and the foetus. Furthermore, as serotonin has a key role in perinatal neurodevelopment, the administration of α-LA in patients with PCOS seeking pregnancy and/or in pregnant women can improve neurological development of the offspring, avoiding the occurrence of consequences later in life.

One of the contemporary challenges of medicine is developing approaches as tailored as possible. In this scenario, strategies that efficaciously modify and re-balance gut microbiota to improve metabolic alterations assume a priority role in the management of a complex and heterogeneous disorder like PCOS. The wide beneficial effect of α-LA paves the way for investigating its therapeutic role in the syndrome, encouraging the association with inositols to enhance their intestinal adsorption.

## Data Availability

Not applicable.
